# Transfemoral amputee recovery strategies following trips to their sound and prosthesis sides throughout swing phase

**DOI:** 10.1186/s12984-015-0067-8

**Published:** 2015-09-09

**Authors:** Camila Shirota, Ann M. Simon, Todd A. Kuiken

**Affiliations:** Department of Biomedical Engineering, Northwestern University, Evanston, IL 60208 USA; Department of Physical Medicine and Rehabilitation, Northwestern University, Chicago, IL 60611 USA; Department of Surgery, Northwestern University, Chicago, IL 60611 USA; Center for Bionic Medicine, Rehabilitation Institute of Chicago, 345 E. Superior St., room 1309, Chicago, IL 60611 USA

## Abstract

**Background:**

Recovering from trips is challenging for transfemoral amputees, and attempts often result in falls. Better understanding of the effects of the sensory-motor deficits brought by amputation and the functional limitations of prosthetic devices could help guide therapy and fall prevention mechanisms in prostheses. However, how transfemoral amputees attempt to recover from trips on the sound and prosthesis sides throughout swing phase is poorly understood.

**Methods:**

We tripped eight able-bodied subjects and eight unilateral transfemoral amputees wearing their prescribed prostheses. The protocol consisted of six repetitions of 6 and 4 points throughout swing phase, respectively. We compared recovery strategies in able-bodied, sound side and prosthesis side limbs. The number of kinematic recovery strategies used, when they were used throughout swing phase, and kinematic characteristics (tripped limb joint angles, bilateral trochanter height and time from foot arrest to foot strike) of each strategy were compared across limb groups. Non-parametric statistical tests with corrections for post-hoc tests were used.

**Results:**

Amputees used the same recovery strategies as able-bodied subjects on both sound and prosthesis sides, although not all subjects used all strategies. Compared to able-bodied subjects, amputees used delayed-lowering strategies less often from 30-60 % of swing phase on the sound side, and from 45-60 % of swing phase on the prosthesis side. Within-strategy kinematic differences occurred across limbs; however, these differences were not consistent across all strategies. Amputee-specific recovery strategies—that are not used by control subjects—occurred following trips on both the sound and prosthesis sides in mid- to late swing.

**Conclusions:**

Collectively, these results suggest that sensory input from the distal tripped leg is not necessary to trigger able-bodied trip recovery strategies. In addition, the differences between sound and prosthesis side recoveries indicate that the ability of the support leg might be more critical than that of the tripped leg when determining the response to a trip. The outcomes of this study have implications for prosthesis control, suggesting that providing correct and intuitive real-time selection of typical able-bodied recovery strategies by a prosthetic device when it is the tripped and the support limb could better enable balance recovery and avoid falls.

**Electronic supplementary material:**

The online version of this article (doi:10.1186/s12984-015-0067-8) contains supplementary material, which is available to authorized users.

## Background

Individuals with transfemoral amputations fall at similar rates to balance-impaired individuals [1, 2], such as the elderly [3] and persons with vestibular dysfunction [4]. An estimated 50 % of amputee falls result in injuries, and 60 % of amputee fallers reported that falls negatively affected their daily activities [5]. Although the causes of falls in transfemoral amputees are often unclear, trips are a major risk as these individuals are unable to adequately recover balance from such perturbations [6]. Patient training has the potential to improve recovery after a trip [7], but requires that the person adapt their movements to a limb replacement with limited mechanical capabilities and that does not adequately replace the functionality of the lost limb [8, 9]. Instead, modifying the response of the prosthesis might help transfemoral amputees avoid falls with potentially less compensatory movements. Newer prosthetic devices can provide net energy to the user [10, 11], which can be used to generate limb movements—capabilities that able-bodied persons use to recover from trips [12, 13].

To date, it is unclear how a knee-ankle prosthesis should respond to trips in order to enable balance recovery. The few studies that investigated amputee tripping [6, 14] provide interesting, albeit insufficient, indications of possible solutions. Simple responses, such as locking the knee joint to avoid buckling when the tripped foot is loaded, are easy to implement but might be overly delayed and insufficient to avoid falls [6]. Alternatively, emulating able-bodied recovery [14] directly replaces the lost limb; however, it is unclear whether, and to what extent, such kinematic responses would still be relevant after the sensory-motor changes brought by amputation.

Successful solutions should be biomechanically appropriate and coordinate with the user’s response. In able-bodied subjects, trip recovery requires short reaction times [15] and good strength [16] of both legs, in addition to quick movements [17] of the tripped leg. Distal sensory inputs from the tripped limb provide information about the nature and location of the perturbation and guide the motor responses necessary to recover from the trip and to re-establish walking [18]. Recovery is typically classified according to the kinematics of the tripped foot as it negotiates the obstacle; able-bodied persons use three distinct kinematic patterns which are strongly related to the onset of the perturbation during swing phase [18, 19]. *Elevating* and *delayed-lowering* strategies are mostly observed in early swing, while *lowering* strategies are typically observed in late swing. During mid-swing, any of these three strategies can be observed and strategy use is further influenced by perturbation duration [20]. How able-bodied subjects select the best strategy to recover balance following a trip is not well understood [18, 21], and it is even less clear how strategy selection would occur in amputees.

Amputation modifies the neuromuscular system by eliminating both the direct sensory input from the amputated limb, as well as the direct control of the motor output of the distal limb segments. The prosthesis establishes indirect sensory-motor paths through its connection to the residual limb. This generally results in asymmetric gait that differs from able-bodied patterns on both prosthesis and sound sides [22, 23]. Coordination between legs is also changed, as reflected by longer sound side stance [24], and preference to lead obstacle crossing with the sound side [25]. Although these known differences may alter the way an individual with a transfemoral amputation perceives a trip and attempts recovery, little is known about how they recover from tripping perturbations. The single previous study that analyzed kinematic recovery patterns in transfemoral amputees suggested that, in mid-swing, amputees might use new kinematic patterns in addition to typical able-bodied strategies [14]. However, limited insight into how recovery strategies were performed is provided. For example, it is unclear how amputees respond to perturbations at other times in swing phase (e.g., when tripped in early or late swing) and how consistently subjects respond to a given tripping perturbation. Understanding how amputees attempt to recover from trips could reveal current limitations in responses of the device, and inform how to best integrate trip recovery or fall prevention functionality in a prosthesis.

Our objective was to understand the effect of transfemoral amputation on the selection and kinematic characteristics of trip recovery strategies needed while attempting to re-establish balance during gait. Based on previous studies, we hypothesized that: (a) When tripped on the sound side, amputees would use the same recovery strategies and at the same onset times as able-bodied subjects; (b) When tripped on the prosthesis side, the lack of direct voluntary knee and ankle flexion in the prosthesis would prevent subjects from using elevating strategies; and (c) When tripped on the prosthesis side, amputees would use other strategies throughout swing phase, resulting from adaptations to the loss of direct sensory feedback from the missing limb and lack of direct control over the prosthesis. Additionally, we expected prosthesis side trips to have increased hip flexion and increased tripped leg height to compensate for decreased prosthetic knee and ankle flexion.

## Methods

### Experimental protocol and data collection

Eight able-bodied subjects (24 ± 2 years old, 1.70 ± 0.07 m, 64.3 ± 9.5 kg, four female) and eight unilateral transfemoral amputees wearing their prescribed prostheses (Table 1) participated in this study. Exclusion criteria for able-bodied subjects included history of leg and/or back injury and neuromuscular disorders. Exclusion criteria for amputee subjects included lower than Medicare K3 level (community) ambulation ability, history of sound leg impairment, presence of co-morbidities that could interfere with the study and any unhealed wounds on the residual limb. All subjects gave informed consent prior to participation in the experiment, which had approval of the local Institutional Review Board.

**Table 1:** Relevant transfemoral amputee subject demographics

Subject	Gender	Age (years)	Time since amputation (years)	Amputated side, Etiology		Residual limb length	Prosthetic knee	Suspension type	Preferred walking speed (m/s)
TF1	Male	50	37.4	Left,	Trauma	Short	3R80	Liner with belt	0.61
TF2	Male	55	43.3	Left,	Trauma	Medium	Mauch	Suction	0.56
TF3	Male	42	19.0	Left,	Trauma	Medium	Total knee	Seal-in liner	0.92
TF4	Female	21	6.1	Left,	Sarcoma	Long	C-Leg	Suction	0.92
TF5	Male	60	6.5	Left,	Trauma	Long	C-Leg	Seal-in liner	0.56
TF6	Male	46	9.6	Left,	Trauma	Long	C-Leg	Seal-in liner	1.0
TF7	Male	53	16.4	Right,	Trauma	Medium	C-Leg	Suction	1.1
TF8	Male	50	16.9	Right,	Trauma	Long	Genium	Pin lock	0.56
Mean ± SD	-	47 ± 12	19.4 ± 13.9	-		-	-	-	0.78 ± 0.23

**Fig. 1:**
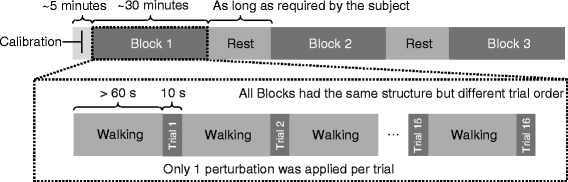
Experimental protocol for transfemoral amputee subjects. The experiment was divided into an undisturbed calibration phase, followed by blocks containing perturbations. In each block, trials of 10 s containing a single perturbation were separated by at least 60 s of walking. A total of 16 trials (4 points in swing phase * 2 repetitions * 2 legs) were recorded per block, in random order. Resting breaks were mandatory between data collection blocks, and could occur throughout the experiment at subjects’ request

To best match the conditions in which subjects are likely to experience trips, distinct but similar protocols (Fig. 1) were used for each subject group. Able-bodied subjects were tripped while walking on a treadmill at the average overground walking speed of 1.4 m/s [26]. Subjects’ feet were attached to retractable tethers that ran through a custom-made tripping device [27]. An initial five minutes of undisturbed walking was used to obtain an estimate of swing phase duration, necessary for the tripping device controller. The remainder of the data were recorded during continuous walking in trials of 10 s separated by at least 1 min. Trips were induced by perturbations that consisted of braking a tether for 150 ms [20]. Perturbations were applied on the right and left sides at six different points in swing phase (10 to 60 % in 10 % increments). Each point was repeated six times, resulting in 72 perturbations per experimental session (6 points * 6 repetitions * 2 legs). This large number of perturbations was chosen to establish a comprehensive reference data set for future studies. In order to avoid fatigue, the experiment was divided in three blocks, each containing 24 perturbations (6 points * 2 repetitions * 2 legs). To avoid anticipatory reactions, trials within each block were randomized. An experimenter conversed with subjects as they were walking to further distract them. Subjects wore a safety harness that did not affect their walking and allowed approximately 15 cm of slack before providing support. Treadmill handrails were also available, although experimenters discouraged subjects from using them. Subjects were allowed as many breaks as necessary during the experiment.

For amputee subjects, the experimental protocol involved two visits. On the first day, subjects were familiarized with the experimental setup and their preferred walking speed was determined. After establishing a comfortable speed, subjects were asked to walk as fast as possible. After five minutes, the treadmill speed was returned to the previous comfortable walking speed, or to a new preferred speed. This was repeated until subjects found a speed they judged they could walk at for blocks of 20 min. We then exposed subjects to a block of perturbations to gauge their comfort with the data collection protocol. On the second day, data were collected as for able-bodied subjects, except that amputees walked at their own preferred speed (0.79 ± 0.23 m/s) and were tripped at 4 different points in swing phase (22 to 67 % in 15 % increments). These protocol changes were made to avoid potential gait modifications caused by subject fatigue or discomfort during walking, given amputees’ slower speed and greater energy expenditure [26].

All data were simultaneously recorded in Cortex (Motion Analysis, Santa Rosa, CA). Motion data from the pelvis and lower limbs [28] were acquired at 100 Hz. Markers on the prosthesis were placed on the knee axis of rotation when possible, and at locations corresponding to landmarks on the sound side [29]. Joint angles were obtained from Visual3D (C-Motion, Germantown, MD). Ground reaction forces from the split-belt force treadmill (ADAL 3D-F/COP/Mz, Medical Developpement, Andrézieux-Bouthéon, France) and load cell data from the tethers were sampled at 1 kHz, and all data were exported to Matlab (The Mathworks, Natick, MA).

### Data analysis

Ground reaction forces were used to detect foot-strike and toe-off. Tether load data were used to determine trip timing. Perturbation onset was re-estimated as the time between toe-off and the trip, normalized by average swing phase duration of each subject, to account for stride variability and possible delays in the tripping device. To verify that we had effectively tripped the subject, we compared the tripped foot’s trajectory (as indicated by the lateral malleolus marker) and swing phase duration to average walking values. Trials for which both variables were within two standard deviations of those for normal walking, indicating ineffective tripping, were not further analyzed. This occurred in two able-bodied trials, nine prosthesis side trips and four sound side trips. Since this trip verification was done in post-processing, these trials were not recollected, resulting in fewer trials for these subjects.

For each valid trip, we identified the recovery strategy used. We then computed kinematic variables to characterize the strategies. Average values for each subject were grouped according to which limb was tripped. For able-bodied subjects, right- and left-side trips were pooled because previous studies indicated no evidence of differences between sides during trip recovery [30]. Thus, we compared data from three tripped limb groups: able-bodied, amputee sound side, and amputee prosthesis side. Details of the analysis are given below.

#### Strategy identification

We identified recovery strategies based on features of the kinematic trajectory of the tripped foot. We compared its position trajectory following the trip to its position when arrested. We used a combination of vertical and anterior-posterior positions of the lateral malleolus of the tripped foot to identify typical able-bodied recovery strategies:

- Elevating strategy: if the foot was raised and placed ahead of the arrested location.
- Delayed-lowering strategy: if the foot was raised and placed at or behind the arrested location.
- Lowering strategy: if the foot was not raised and placed at or behind the arrested location.
- Incomplete arrest: if the foot was not raised and placed ahead of the arrested location.

After trips, control subjects maintain a reciprocal stepping pattern in which stepping order and foot placement ahead of the body are alternated between sides. However, we expected that this might not always occur in transfemoral amputees [14]. Using amputees’ stepping pattern after a trip, we identified two amputee-specific recovery strategies.

- Hopping strategy: the alternating stepping pattern was interrupted and the subject jumped over the virtual obstacle with both feet in the step following the trip (i.e., bringing both feet forward during a flight phase).
- Skipping strategy: the alternating stepping pattern was interrupted and the subject took an extra step with the tripped foot prior to moving the support foot. In this new strategy, the tripped foot was quickly lowered after the trip and was the first to overcome the virtual obstacle.

To assess the variety of recovery strategies elicited, we compared the average number of strategies used per subject for each tripped limb group.

#### Kinematics of typical able-bodied recovery strategies

To further understand the limitations imposed by the prosthesis on the tripped foot trajectory that characterizes each recovery strategy, we compared the maximum foot elevation and anterior foot placement with respect to its arrested position. We were also interested in understanding how amputees achieved typical able-bodied strategies without active knee and ankle joints on the prosthesis side. For this, we compared time elapsed from tripped foot arrest to foot-strike, and maximum hip, knee and ankle joint flexion angles on the tripped leg relative to the angles at foot arrest [20]. In addition, we quantified the use of other compensatory mechanisms to raise the tripped leg by measuring the vertical displacement of the proximal end of the thigh (trochanter) on the tripped and the support legs. We did this by averaging the difference between recovery and walking trochanter height from foot arrest to foot-strike.

#### Recovery strategy distribution throughout swing phase

The incidence of the different strategies throughout swing phase was compared across tripped limb groups to assess the effect of trip onset on strategy selection. Trials from 15-75 % of swing phase were separated into bins representing 15 % increments of swing phase in order to compare the able-bodied and amputee data. For each subject, the relative occurrence of each recovery strategy was calculated in each of the four bins.

#### Statistics

Non-parametric tests were used due to the non-Gaussian nature of the data, and the limited number of points per group. Rank-sum tests were used to compare able-bodied to amputee sound side and prosthesis side data. Sign-rank tests were used to compare results between the sound and prosthesis sides, since the same subjects are measured in both conditions. Results were considered significant at the 0.05 level, which corresponds to 0.0167 per comparison with a Bonferroni correction.

## Results

### Recovery strategies

We observed six distinct kinematic patterns in response to tripping perturbations (Table 2; see video in Additional file 1). The four typical able-bodied strategies were elicited in all limb groups, although not all subjects employed all strategies. For each amputee, we compared the sets of typical able-bodied strategies elicited on their sound and prosthesis sides. For six out of eight subjects, the set of (typical able-bodied) strategies they used when tripped on their sound side was a subset of the (typical able-bodied) strategies they used on their prosthesis side. The remaining two subjects behaved similarly, except that their set of sound side strategies contained incomplete arrests in addition to strategies they also used on the prosthesis side. All able-bodied and amputee subjects used the lowering strategy on both sides. Four amputee subjects used amputee-specific strategies (hopping and skipping) when tripped on the sound side, and two subjects used these strategies (hopping) when tripped on the prosthesis side. Although the average number of strategies used per subject was smaller on the sound side, this difference did not reach significance (able-bodied compared to sound side: *p* = .02; able-bodied compared to prosthesis side: *p* = .31; sound side compared to prosthesis side: *p* = .5).

**Table 2:** Number of subjects that used each recovery strategy

Tripped limb	Elevating	Delayed-lowering	Lowering	Incomplete arrest	Skipping	Hopping	Average number of strategies per subject
Able-bodied	6	8	8	8	0	0	3.8
Sound side	3	1	8	6	3	2	2.9
Prosthesis side	5	6	8	6	0	2	3.4

Typical able-bodied strategies are elevating, delayed-lowering and lowering strategies, and incomplete arrests

Amputee-specific strategies are skipping and hopping

### Kinematics of typical able-bodied recovery strategies

**Fig. 2:**
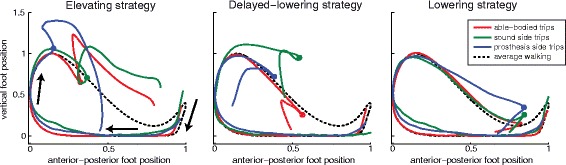
Sagittal positions of the tripped foot throughout strides representative of recovery strategies during treadmill walking. Trajectories of the tripped foot during able-bodied (*red*), amputee sound side (*green*) and amputee prosthesis side (*blue*) recoveries are shown during elevating (*left*), delayed-lowering (*middle*) and lowering (*right*) strategies. Undisturbed walking of the able-bodied subject is also illustrated for reference (*dashed line*); note that differences in trajectories before the trip occurs are due to different baseline trajectories and not early trajectory deviations. Arrows indicate the direction of foot movement throughout a gait cycle from foot strike to the subsequent foot strike. Positions are defined with respect to a global reference frame. Hence, the foot moves posteriorly during stance, due to movement of the treadmill belt. Dots indicate where the foot was arrested in trip trials. Relative to the position at foot arrest, there were no significant differences across limbs in the elevating strategy. In delayed-lowering strategies, the prosthetic foot was elevated more than in able-bodied recoveries. Lowering strategies on the prosthesis side were achieved with less foot elevation than in able-bodied subjects. Vertical and horizontal axes were scaled to the undisturbed walking amplitude for each subject. Horizontal (*vertical*) foot position of each stride was zeroed at its most posterior (*inferior*) position

For each recovery strategy, foot trajectories were similar across limb groups (Fig. 2). We observed no significant differences in tripped foot kinematics (as measured by the elevation and anterior placement of the foot relative to its position when arrested) between sound side in amputees and able-bodied limbs, or sound side and prosthesis side in amputees. However, post-trip, amputees raised their prosthetic foot more than able-bodied subjects during delayed-lowering strategies (*p* = .008) and less during lowering strategies (*p* = .0003).

**Fig. 3:**
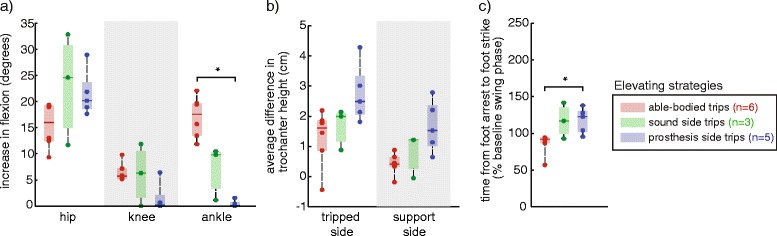
Comparison of kinematic characteristics for elevating strategies across able-bodied, sound side and prosthesis side trips. **a** Increases in joint flexion (ankle dorsiflexion) were measured relative to the joint angles at trip onset. Elevating strategies on the prosthesis side had statistically smaller increases in the ankle dorsiflexion compared to able-bodied subjects (*p < .05, familywise). **b** There were no differences in trochanter height across tripped limb groups. **c** Elevating strategies were statistically longer in duration for prosthesis side trips in amputees than in able-bodied subjects. Dots indicate individual subject averages. Number of subjects per group is indicated in the legend

Elevating strategies (Fig. 3) on the prosthesis side had statistically longer durations in amputees than in able-bodied subjects (*p* = .004), with smaller changes in ankle dorsiflexion (*p* = .004) (as expected due to subjects’ rigid prosthetic ankles). Sound side recovery was not statistically different from prosthesis side or able-bodied recovery.

**Fig. 4:**
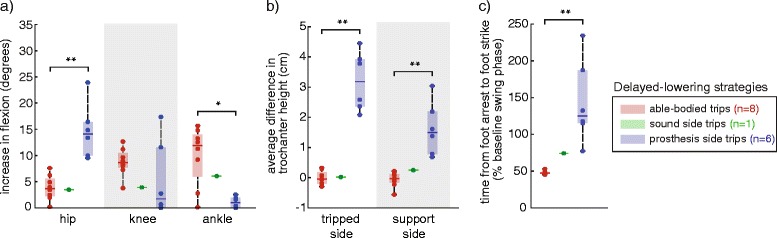
Comparison of kinematic characteristics of delayed-lowering strategies across able-bodied, sound side and prosthesis side trips. **a** Increases in joint flexion (ankle dorsiflexion) were measured relative to the joint angles at trip onset. Delayed-lowering strategies on the prosthesis side had statistically larger increase in hip flexion, and smaller increase in ankle dorsiflexion, compared to able-bodied subjects (*p < .05, **p < .005, familywise). **b** Prosthesis side delayed-lowering strategies had a higher average difference in greater trochanter height on the tripped and sound sides compared to able-bodied subjects. **c** Recovery on the prosthesis side required more time than in able-bodied subjects

Delayed-lowering strategies (Fig. 4) on the prosthesis side had statistically decreased ankle dorsiflexion (*p* = .012) and increased hip flexion (*p* = .0007), with increased greater trochanter vertical displacement on both the tripped (*p* = .0007) and support sides (*p* = .0007) than in able-bodied subjects. Prosthesis side delayed-lowering strategies also required more time to complete than able-bodied delayed-lowering strategies (*p* = .0007). On the sound side, a single subject used delayed-lowering strategies; the kinematic characteristics were more similar to able-bodied recovery than to prosthesis side delayed-lowering strategies.

**Fig. 5:**
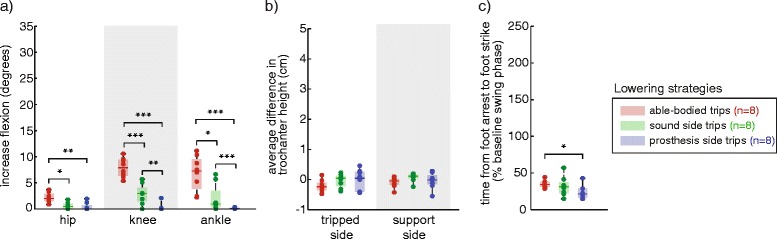
Comparison of kinematic characteristics of lowering strategies across able-bodied, sound side and prosthesis side recoveries. **a** Increases in joint flexion (ankle dorsiflexion) were measured relative to the joint angles at trip onset. Lowering strategies on the prosthesis and sound sides were achieved with smaller peak hip, knee and ankle flexion post-trip than in able-bodied subjects (*p < .05, **p < .01, ***p < .005, familywise). Sound side lowering strategies involved increased knee and ankle flexion compared to the prosthesis side. **b** There were no differences in trochanter height across tripped limb groups. **c** Prosthesis side lowering strategies were shorter than in able-bodied subjects

Lowering strategies (Fig. 5) on the prosthesis side had statistically smaller increases in ankle dorsiflexion (*p* = .00016), knee flexion (*p* = .00016) and hip flexion (*p* = .0028), and had shorter duration (*p* = .015) than in able-bodied subjects. Sound side lowering strategies had statistically more ankle dorsiflexion (*p* = .0016) and knee flexion (*p* = .0078) than on the prosthesis side. In comparison to able-bodied limbs, sound side lowering strategies had smaller increases in ankle dorsiflexion (*p* = .0047), knee flexion (*p* = .0006) and hip flexion (*p* = .0047).

### Recovery strategy distribution throughout swing phase

**Fig. 6:**
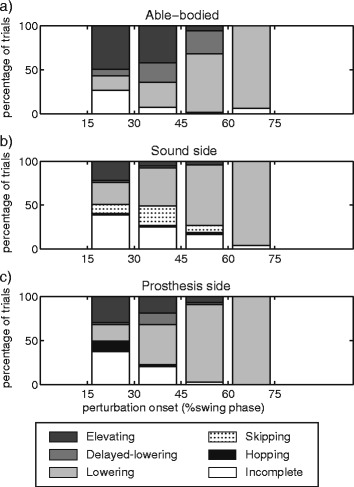
Relative frequency of recovery strategies throughout swing phase. **a** Able-bodied subjects used elevating and delayed-lowering strategies in early to mid-swing. As onset increased, lowering strategies were increasingly employed. **b** For sound side limbs, recovery from trips was achieved using typical able-bodied strategies in the same intervals of swing phase as able-bodied subjects, although the elevating and delayed-lowering strategies were less frequently used for trips from 30-45 % and 30-60 % of swing phase, respectively. Skipping and hopping strategies were used in early to mid-swing. **c** Trips on the prosthesis side elicited typical able-bodied recovery strategies, but amputees used delayed-lowering strategies less than able-bodied subjects from 45-60 % of swing phase. Hopping strategies were used in early to mid-swing

In amputees, the type of recovery strategy used was related to perturbation onset (Fig. 6). When amputee subjects used typical able-bodied recovery strategies, they did so in the same intervals of swing phase as able-bodied subjects. However, compared to able-bodied subjects, amputees used delayed-lowering strategies less frequently in response to trips occurring from 30-60 % of swing phase on the sound side (30-45 %: *p* = .004; 45-60 %: *p* = .002) and from 45-60 % of swing phase on the prosthesis side (*p* = .0006). Hopping strategies were used in response to perturbations in early and mid-swing on the prosthesis and sound sides, and skipping strategies were used in early to mid-swing on the sound side.

## Discussion

The current study is an investigation of the kinematics of transfemoral amputee recovery from tripping perturbations throughout swing phase on the prosthesis and sound sides. When provided with balance support, amputees used typical able-bodied (i.e., elevating, delayed-lowering, and lowering) and amputee-specific (i.e. hopping and skipping) recovery strategies in response to trips on both sides (Table 2). However, the distribution of trip recovery strategies throughout swing phase on either side was different from able-bodied subjects (Fig. 6). Kinematic characteristics of typical able-bodied strategies in amputees revealed bilateral compensations for unilateral passive knee and ankle joints (Figs. 3, 4 and 5). These results support the idea that recovery from perturbations during gait involves complex sensory-motor coordination between the tripped and support legs. Their roles are further elucidated by the adaptations we observed to the abilities of the sound and prosthesis sides of transfemoral amputees – as discussed in the following paragraphs.

Our hypotheses – that responses to trips on the sound side would resemble those of control subjects, while reactions to trips on the prosthesis side would not – were based on the assumption that responses to trips are strongly dependent on the ability of the tripped leg to i) sense the perturbation, and ii) perform the kinematic patterns associated with each recovery strategy. However, sensory-motor integrity – as represented by the intact sound limb and the deficient prosthesis limb – did not determine which strategies were used by amputees. In the motor aspect, deficits of the tripped prosthesis side did not impede amputees from using typical able-bodied strategies, and neither did an intact tripped leg respond as an able-bodied limb. On the prosthesis side, we did not expect amputees to use the elevating strategy, since it requires ankle and knee flexion on the tripped side to achieve clearance of the foot over the obstacle. Instead, a fairly large portion of the amputee subjects used it at least once (Table 2), with end-point (foot) trajectories that were similar to those from able-bodied subjects (Fig. 2). To achieve these foot trajectories, decreased prosthesis ankle and knee movement were compensated by increased residual hip flexion and bilateral raising of the pelvis during a prolonged recovery period (Fig. 3). Although only the latter was statistically different, these kinematic changes are analogous to deviations typical of amputee gait to ensure prosthetic foot clearance, such as vaulting and hip-hiking [9]. Similar compensatory movements were observed in delayed-lowering strategies (Fig. 4). Throughout swing phase, this resulted in near-normal (able-bodied) use of recovery strategies for the tripped prosthetic limb (Fig. 6). In contrast, such kinematic differences were not evident on the sound side data, which tended to be within the range of able-bodied values or between able-bodied and prosthesis side values (Figs. 3 and 4). However, these near-normal kinematics of sound side trip recovery were accompanied by slightly more changes to strategy distribution throughout swing phase than for the tripped prosthetic limb (Fig. 6). Further, the number of subjects that used sound side elevating and delayed-lowering strategies is fewer even than on the prosthesis side (Table 2). This difference to able-bodied behavior is likely not due to a simple shift in the response throughout swing phase caused by, e.g., delayed sensory feedback or delays in sound side responses [31]. Alternatively, it has been shown that the support leg contributes to balance recovery by actively restraining the body's forward angular momentum before tripped foot-strike [32]. It is likely difficult to produce counter-acting moments with a passive knee and rigid ankle, i.e. when the prosthesis is the support leg. In contrast, this can still be achieved by the sound leg when the prosthetic leg is tripped. Thus, having an intact tripped limb is not sufficient, nor is it required, to enable recovery as in able-bodied subjects. Instead, our results suggest that recovery depends on good bilateral coordination and may be more influenced by the ability of the support leg to respond to perturbations to the swing leg.

The differences between tripped sound and prosthesis limbs also contribute to our understanding of the neural mechanisms underlying the selection of a recovery strategy. In able-bodied subjects, localized sensory input from the tripped foot and distal leg contribute to widespread (multi-joint and bilateral) and generic short latency responses to perturbations during gait [33]. Functional, longer latency components likely involve a combination of various sensory inputs, possibly through neural circuits involving the brain [34]. Strategy distribution on the prosthesis side was similar to able-bodied subjects (Fig. 6), despite the lack of direct input from the distal tripped leg. On the other hand, intact distal tripped leg information did not result in the use of strategies as seen in able-bodied subjects. This suggests that, although mechanical and electrical (cutaneous) stimuli on the distal leg can elicit trip recovery strategies [35, 36], direct sensory feedback is not necessary - nor does it imply - recovery as in able-bodied subjects. Indeed, amputees have to adapt to the new sensory feedback. This agrees with neural adaptations following amputation that have been reported in the literature [37, 38]. However, it has also been suggested that sensory feedback from the support leg - specifically the center of pressure - is important to control gait and trip recovery strategies [39]. This could be an alternative explanation to the strategy distributions we observed throughout swing phase.

Although sensory input in amputees was not the same as in able-bodied subjects, the responses elicited were consistent within subjects. We assessed consistency by observing how many times a strategy was used in response to multiple instances (repetitions) of a given perturbation. For every amputee subject, we found at least one inconsistent response, i.e., one strategy that was used a single time in an onset window. However, these single-occurring responses were consistent with responses from other amputees, except for the rarely used delayed-lowering strategy. Additionally, the timing of these single responses throughout swing phase agreed with able-bodied data. Thus, we interpret these apparently inconsistent behaviors as patterns that would be observed consistently in studies involving more repetitions of each perturbation than in this study. Strategy use across subjects was more variable than within-subject, especially in the number of subjects that used each strategy, which is likely due to the heterogeneity of our study population.

These results suggest that emulating able-bodied responses when the prosthesis is tripped would likely agree with subjects’ intentions. Such responses have the potential to better replace the lost limb, without requiring compensatory movements. On the other hand, our results indicate that it is also important to implement adequate responses when the prosthesis is the support limb, as its ability to coordinate with the tripped leg greatly influences how subjects recover balance.

### Study limitations

The possibility of using handrails or the harness for support could have affected subjects’ response to perturbations since the sense of stability provided by light touch on handrails is enough to alter responses to unbalancing perturbations [40]. In our study, this was necessary to allow amputees to complete recovery from the trip, providing original data on the response considered most adequate by their body at the time of the external perturbation, instead of completely, and repeatedly, falling down. Another potential limitation is that subjects were not matched across groups. Although increased age has been associated with decreased trip recovery abilities [41, 42], we assumed that the impairments brought by transfemoral amputation [14] would overshadow those due to age [43]. Future studies could investigate their relative contributions. Our attempt at emulating trips in a realistic context resulted in lower walking speeds in the amputee group than in controls. Increasing walking speed in amputees would likely hinder subject participation and their ability to complete the protocol by, for example, inducing early fatigue and discomfort within the socket. Alternatively, decreasing control subjects’ walking speed would have likely increased the incidence of elevating strategies [43], further increasing the differences we observed between subject groups. We believe that allowing amputees to ambulate at their self-selected speed best reflects the challenge of recovering from trips in a comparable context to trips in control subjects. Finally, our amputee population was relatively small and heterogeneous. Most surprisingly, differences in prosthetic knee did not correspond to differences in results across subjects, despite the range in their capabilities. Such trends were also not observed for walking speed, time since amputation and residual limb length. Additionally, we maintained each subject’s prescribed prosthesis settings in order to observe the most representative real-world responses to tripping perturbations. Future studies investigating the mechanical settings of the prostheses, which influence the device’s behavior, could further differentiate how amputees respond to perturbations.

## Conclusion

Transfemoral amputees generally exhibited typical able-bodied recovery strategies (e.g., elevating, lowering, delayed-lowering) when recovering from trips on both the sound and prosthesis sides with balance support. Throughout swing phase, amputees used similar recovery strategies to able-bodied subjects for perturbations that occurred at similar time points in the gait cycle, although delayed-lowering strategies were used less frequently in mid-swing. In addition, we observed novel strategies – hopping on the prosthesis side and skipping on the sound side – unique to amputees following trips in early to mid-swing on both sides. Changes to the sensory-motor system due to transfemoral amputation affected recovery from trips of both the prosthesis and sound sides, with the largest changes in strategy selection on the sound side. This suggests that the role of the stance leg is critical to trip recovery. Therefore, prosthetic fall prevention mechanisms capable of robustly executing elevating, delayed-lowering and lowering strategies as both the tripped and support legs would be beneficial in providing natural and intuitive recovery from tripping perturbations in transfemoral amputees.

## Additional file

Additional file 1:
**Representative examples of recovery strategies in response to trips on able-bodied, prosthesis and sound side limbs.** Graphical representation of able-bodied and amputee subjects recovering from trips. The video is separated by recovery strategies; sample trials from each limb group that presented a specific recovery strategy are presented in sequence. Each trial begins with foot-strike of the perturbed stride and continue for one or two additional strides for typical able-bodied and amputee-specific strategies, respectively. The tripped leg is highlighted in red. The pelvis and lower limbs are represented by bony segments (intact limbs) or cylindrical segments (prosthesis side). Video speed is reduced 2.5 times. (MP4 18472 kb)
